# The secreted peptide BATSP1 promotes thermogenesis in adipocytes

**DOI:** 10.1007/s00018-023-05027-9

**Published:** 2023-11-27

**Authors:** Xianwei Cui, Hong Zhong, Yangyang Wu, Zhuo Zhang, Xiaoxiao Zhang, Lu Li, Jin He, Chen Chen, Zhenggang Wu, Chenbo Ji

**Affiliations:** Nanjing Maternal and Child Health Institute, Women’s Hospital of Nanjing Medical University, Nanjing Maternity and Child Health Care Hospital, Nanjing, 210004 Jiangsu China

**Keywords:** Peptidome, Secretion, Energy expenditure, Phosphorylation, Translocation

## Abstract

Although brown adipose tissue (BAT) has historically been viewed as a major site for energy dissipation through thermogenesis, its endocrine function has been increasingly recognized. However, the circulating factors in BAT that play a key role in controlling systemic energy homeostasis remain largely unexplored. Here, we performed a peptidomic analysis to profile the extracellular peptides released from human brown adipocytes upon exposure to thermogenic stimuli. Specifically, we identified a secreted peptide that modulates adipocyte thermogenesis in a cell-autonomous manner, and we named it BATSP1. BATSP1 promoted BAT thermogenesis and induced browning of white adipose tissue in vivo, leading to increased energy expenditure under cold stress. BATSP1 treatment in mice prevented high-fat diet-induced obesity and improved glucose tolerance and insulin resistance. Mechanistically, BATSP1 facilitated the nucleocytoplasmic shuttling of forkhead transcription factor 1 (FOXO1) and released its transcriptional inhibition of uncoupling protein 1 (UCP1). Overall, we provide a comprehensive analysis of the human brown adipocyte extracellular peptidome following acute forskolin (FSK) stimulation and identify BATSP1 as a novel regulator of thermogenesis that may offer a potential approach for obesity treatment.

**Supplementary Information:**

The online version contains supplementary material available at 10.1007/s00018-023-05027-9.

## Introduction

Brown adipose tissue (BAT) is known to dissipate energy via uncoupling protein 1 (UCP1)-mediated nonshivering thermogenesis [1]. Thus, boosting brown fat activity leads to reduced adiposity, improved insulin action and glucose handling, contributing to protection against obesity and metabolic dysfunction [2]. In addition to its function as a “stove” that burns fat, the secretory role of BAT in maintaining whole-body energy homeostasis has recently been recognized [3]. To date, an increasing number of factors secreted from BAT have been identified in explorations of thermogenic stimuli, and these secreted factors exert autocrine, paracrine, and endocrine effects [4]. Some of these signaling molecules are endocrine factors that facilitate communication between adipose tissue and other organs, such as the liver. Recent work has demonstrated that neuregulin 4 (Nrg4), a brown fat-enriched adipokine, rectifies diet-induced obesity and fatty liver by alleviating hepatic lipogenic signaling [5]. In addition, some brown adipokines have been reported to have local actions and target brown adipocytes to increase energy expenditure. For example, ependymin-related protein 1 (EPDR1) is a novel batokine that regulates thermogenesis and influences overall energy consumption [6]. Therefore, the secretory function of BAT is currently recognized as a nonnegligible mechanism by which it exerts its effects, especially in an endocrine manner, but tremendous efforts are still needed to comprehensively understand the BAT secretome.

Peptide hormones have profoundly impacted systemic energy homeostasis and have contributed significantly to the development of biological therapeutics for metabolic diseases such as obesity, diabetes, and cardiovascular diseases [7]. There is no better example to open our discussion than the discovery and development of glucagon-like peptide 1 (GLP-1), which is known not only for its glycemic benefits but also for its weight-lowering efficacy via inhibition of food intake and elevation of energy expenditure [8]. Another example of an endogenous peptide derived from proglucagon and exhibiting an anti-obesity effect is glucose-dependent insulinotropic polypeptide (GIP) [9]. Administration of acyl-GIP leads to reductions in body weight and food intake, along with improved glucose handling. Over the years, a few peptides secreted from adipocytes have been characterized that often perform their actions through actuating thermogenesis in BAT or inducing browning of white adipose tissue (WAT). Irisin, a cleaved and secreted fragment of Fndc5, has been proposed to increase UCP1 expression and cause an increase in total body energy expenditure and resistance to diet-induced obesity [10]. However, some issues remain unresolved, including the controversial role of irisin in human metabolism, the unknown proteolytic enzyme, the poorly vetted and nonspecific detection method, etc. [11]. Such batokines with biological activity are thus far largely undiscovered, and a comprehensive mapping of peptides secreted from BAT is needed.

Recently, the peptidomic approach, which enables quantitative and systematic profiling of peptides in various tissues and fluids, has attracted increasing attention [12]. To identify novel peptides that may contribute to energy expenditure in brown adipocytes, we analyzed the extracellular peptidome of human brown adipocytes and identified BATSP1 (BAT-secreted peptide 1) as an inducible batokine that responds to thermogenic activation. BATSP1 acts as a peptide hormone that promotes adipose thermogenesis both in vitro and in vivo. Furthermore, BATSP1 reduces high-fat diet (HFD)-induced obesity and improves glucose metabolism and insulin sensitivity upon mild cold exposure. The underlying mechanism involves the upregulation of FOXO1 phosphorylation, which in turn regulates the translocation of FOXO1 from the cytosol to the nucleus and alleviates its suppression of UCP1 transcription. Thus, the identification of BATSP1 provides a novel therapeutic strategy for obesity and a basis for peptide drug development.

## Materials and methods

### Primary adipocyte cultures and differentiation

Human primary brown adipocytes (hBA) and white adipocytes (hWA) were isolated and differentiated in medium as previously described [13]. For differentiation of hBA, confluent cells were cultured in DMEM/F12 containing 100 nM insulin, 1 μM rosiglitazone, 0.5 mM isobutylmethylxanthine, 1 μM dexamethasone, 10 μg/mL apo-transferrin and 1 nM T3. After 3 days, the medium was replaced with maintenance medium (100 nM insulin and 1 nM T3 in DMEM/F12). For differentiation of hWA, adipogenesis was initiated by culture in DMEM/F12 medium with 0.5 mM isobutylmethylxanthine, 1 μM dexamethasone, 500 nM insulin and 1 μM rosiglitazone for 4 days. Then, the cells were transferred to medium containing 500 nM insulin, and the medium was replenished every 2 days until Day 8. For BATSP1 treatment, synthesized BATSP1 (Science peptides, Shanghai, China) or vehicle control was added to the medium of differentiated adipocytes, and the cells were incubated for 6 h.

### Respiration assays

Cellular oxygen consumption was assessed using a Seahorse XF24 Analyzer (Agilent Technologies, USA) as previously described [14]. Briefly, primary adipocytes were seeded and differentiated in a Seahorse cell culture plate. The differentiated adipocytes were washed once with prewarmed PBS and cultured with BATSP1 in XF base medium (Agilent Technologies, USA) supplemented with 25 mM glucose, 1 mM pyruvate and 2 mM l-glutamine (pH 7.4). Cells were then subjected to the mitochondrial stress test by the addition of oligomycin (5 µM) followed by FCCP (5 µM) and antimycin (5 µM). To investigate the effect of FOXO1 on cellular oxygen consumption, primary adipocytes were infected with adenoviruses encoding the full-length human FoxO1 gene (HANBIO, Shanghai, China) before bioenergetic profiling. Seahorse XF24 software automatically calculated the oxygen consumption rate (OCR).

### Temperature measurements

Skin temperatures were recorded with an infrared camera (Thermo GEAR G120/G100, NEC Avio Infrared Technologies Co., Ltd., Tokyo, Japan). To induce cold stress, mice were transferred from a 25 °C environment to a 4 °C environment with free access to water and food. The rectal temperature was measured using a BAT-12 microprobe thermometer (Physitemp Instruments Inc., Clifton, NJ, USA) at the indicated time points.

### Dual-luciferase reporter assay

The 4148-bp UCP1 promoter region was cloned from pLightSwitch_hUCP1-Prom (S723122; Switch Gear Genomics, Carlsbad, CA) and inserted into the pGL3-basic plasmid (Promega). Subconfluent HEK 293 T cells were infected with adenoviruses for overexpression of human FoxO1. The cells were then transiently cotransfected with pGL3-Ucp1 and pRL-TK (Promega) in the presence or absence of BATSP1. Firefly and Renilla luciferase activities were measured in cell lysate aliquots using the Dual-Luciferase Reporter System (Promega) according to the manufacturer’s instructions.

### Study in mice with diet-induced obesity

Male mice on a C57BL/6 background were purchased from the Animal Core Facility of Nanjing Medical University and housed on a 12/12 h light/dark cycle with free access to food and water. Mice were maintained in cages exposed to room air (25 °C) or in cages inside temperature-controlled chambers (16 °C) as indicated in the figure legend. After acclimation to the facility (1 week), mice were fed a high-fat diet with 60% of the calories from fat (Research Diets) starting at 7–8 weeks of age to induce obesity. Synthesized BAPSP1 or vehicle (saline) was administered by intraperitoneal (i.p.) injection twice a week for 16 weeks. Body weight and food intake were monitored daily. The total amounts of fat and lean mass in HFD-fed mice were assessed with a nuclear magnetic resonance imaging (MRI) system (Minispec mq 7.5, Bruker Optics).

### Metabolic cage study

Indirect calorimetry was performed on mice receiving 2 weeks of injections of BATSP1 or vehicle at the Animal Core Facility of Nanjing Medical University. Mice were housed individually on a 12/12 h light–dark cycle with the lights on from 7:00 to 19:00. We monitored the mice for 72 h in metabolic cages (TSE systems GmbH, Bad Homburg, Germany) at 25 °C with ad libitum access to food and water. Carbon dioxide production, O_2_ consumption, heat generation, total locomotor activity and food intake were determined, and the parameters were calculated for each mouse according to body weight.

### Glucose tolerance test (GTT) and insulin tolerance test (ITT)

For the GTT, mice fasted overnight (16 h) were injected intraperitoneally with glucose at a dose of 2 g/kg body weight. For the ITT, mice were fasted for 6 h and were then injected intraperitoneally with insulin at 0.75 U/kg body weight. Blood glucose concentrations were measured at the time of and 15, 30, 60, 90 and 120 min after glucose or insulin injection.

### Micro PET/CT

PET/CT imaging was performed with a small animal PET/CT system (Siemens Medical Solution, Germany). After injection of BATSP1 for the indicated time, mice were fasted overnight and lightly anesthetized with isoflurane prior to tail vein administration of ^18^F-fluorodeoxyglucose (^18^F-FDG). Ten-minute static PET scans were acquired beginning 30 min post-injection, and then CT images were analyzed 40 min after ^18^F-FDG injection. The PET data were reconstructed using a 3D ordered subset expectation maximization (OSEM3D) algorithm with CT-based attenuation and scatter correction. ^18^F-FDG uptake was calculated by manually circling regions of interest over areas containing BAT according to the CT images. ^18^F-FDG uptake was expressed as the percentage of injected dose per gram of tissue (%ID/g).

### Imaging experiments

Mice were injected intraperitoneally with N-terminal fluorescein isothiocyanate (FITC)-labeled BATSP1 at a dose of 50 mg/kg body weight. Three hours later, the mice were anesthetized with isoflurane, and fluorescence parameters were measured using an IVIS Spectrum Imaging System (Xenogen, Alameda, CA, USA). The accumulation of FITC-labeled BATSP1 was visualized in excised tissues, including the heart, liver, spleen, kidney, inguinal WAT (iWAT), epididymal WAT (eWAT), BAT and muscle.

### Transmission electron microscopy (TEM)

Sections from BAT and iWAT were fixed with 2.5% (vol/vol) glutaraldehyde in 100 mM phosphate buffer (pH 7.2–7.4) overnight at 4 °C. The samples were then postfixed in 1% osmium tetroxide, dehydrated in an alcohol series (30, 50, 80, 90, and 100%) for 2 h and embedded in epoxy resin. Ultrathin sections with a thickness of 60–80 nm were obtained, placed on formvar/carbon-coated copper grids and stained with lead citrate before visualization in an EM-100S TEM (JEOL, Tokyo, Japan).

### Subcellular fractionation

Human primary brown and white adipocyte cultures were prepared, and BATSP1 was added to the culture medium for 3 h. Cells were then collected, washed once in PBS and placed on ice. Five hundred microliters of fractionation buffer from the PARIS kit (Thermo Fisher Scientific) was used to resuspend the cells, and the suspension was incubated on ice for 10 min. Samples were centrifuged at 500×*g* and 4 °C for 5 min, and then the cytoplasmic fraction was carefully aspirated from the nuclear pellets. The nuclear pellet was further lysed in Cell Disruption Buffer before processing the sample for Western blotting. HSP90 and Lamin B1 were used as markers for the cytosol and nucleus, respectively.

## Results

### Identification of BATSP1 as a regulator of adipocyte thermogenesis

**Fig. 1:**
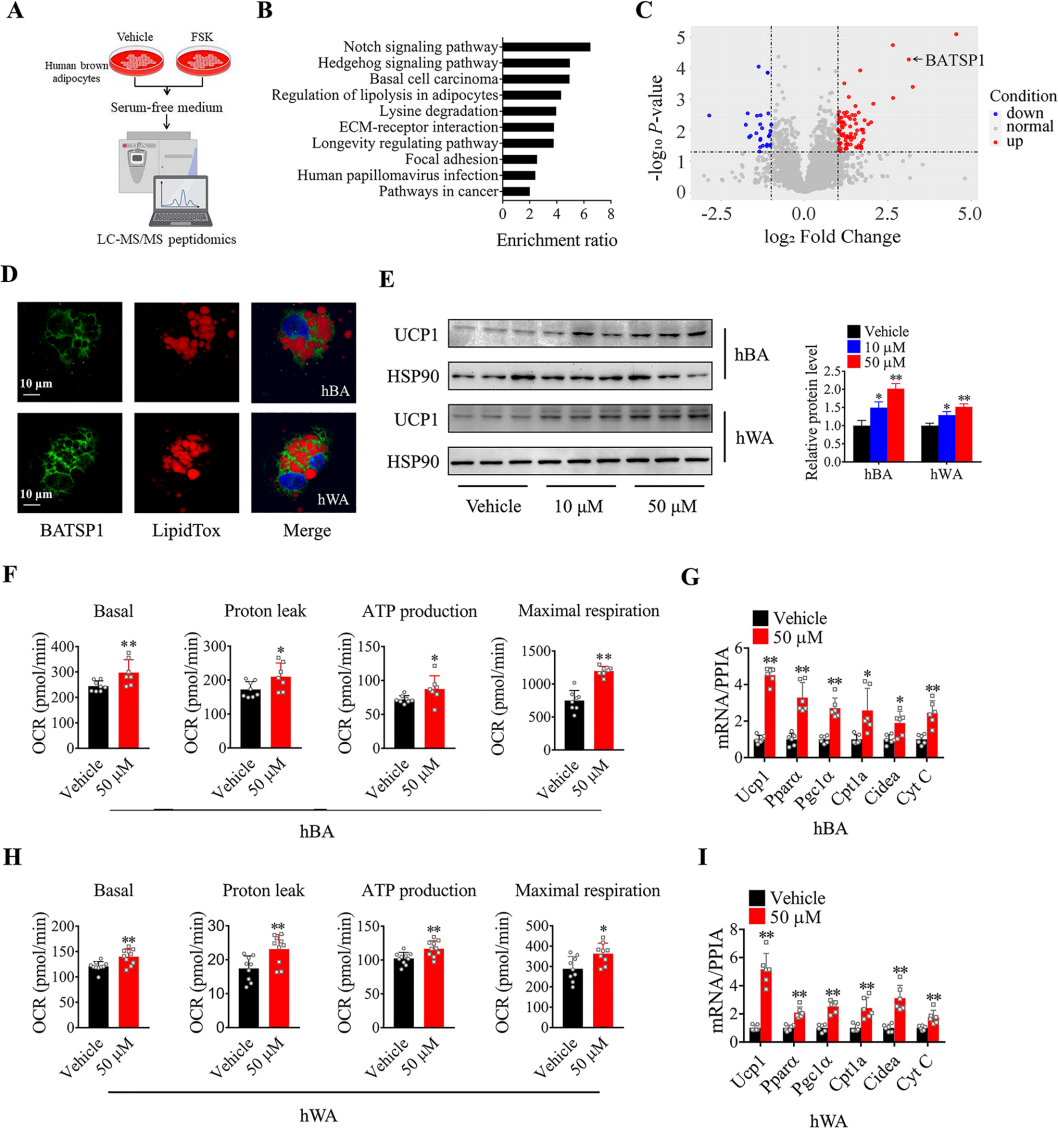
Identification of BATSP1 as a regulator of adipocyte thermogenesis. **A** Flow chart showing the search strategies used to identify human brown adipocyte-secreted peptides. **B** Top KEGG pathways significantly enriched in the precursor proteins identified from the dysregulated peptides. **C** Volcano plot depicting differentially expressed extracellular peptides in brown adipocytes treated with either FSK or vehicle control. **D** Representative confocal images of adipocytes treated with FITC-labeled BATSP1. Cells were costained with LipidTox and DAPI. **E** Western blot analysis of UCP1 expression in fully differentiated brown and white adipocytes after BATSP1 stimulation for 6 h. **F** Basal OCR, proton leakage, ATP production and maximal respiration in BATSP1-treated brown adipocytes. **G** Thermogenic gene expression in brown adipocytes. **H**, **I** Cellular respiration and thermogenic gene expression in white adipocytes were examined by a Seahorse analyzer and real-time quantitative PCR (RT‒qPCR), respectively, after BATSP1 treatment. hBA, human brown adipocytes; hWA, human white adipocytes. The data are presented as the means ± SDs; *p < 0.05; **p < 0.01 by unpaired Student’s *t* test

Previously, we successfully isolated human primary brown adipocytes and optimized the differentiation conditions [13]. To identify extracellular peptides responsible for thermogenesis, we stimulated mature human brown adipocytes with forskolin (FSK) for 4 h. Serum-free conditioned medium was then collected to extract peptides and further sent for liquid chromatography-tandem mass spectrometry (LC‒MS/MS) analysis (Fig. 1A). A total of 4015 secreted peptides derived from 1322 protein precursors were identified in both groups (Table S1). Of these peptides, 357 showed a significant difference in abundance, with a fold change of > 1.3 and a p value of < 0.05 (*t* test), namely, 236 peptides with an increased abundance and 121 peptides with a decreased abundance upon FSK treatment (Table S2). We then analyzed the general features of these peptides. Most of the peptides had a molecular weight of between 0.4 and 1.6 kDa and had an acidic isoelectric point (pI) range (3.0–6.0) (Fig. S1A). A typical characteristic of this set of identified peptides was the presence of 15 peptides derived from the Mucin 16 (MUC16) protein. The top 20 precursor proteins from which the highest number of related peptides were derived are listed in Fig. S1B. Many of these precursors have known biological functions in pathways such as the notch signaling pathway, hedgehog signaling pathway, basal cell carcinoma, and regulation of lipolysis in adipocytes (Fig. 1B). The 25 peptides with the greatest increases and decreases in abundance, as determined by the highest fold changes, were visualized in heatmaps (Fig. S1C).

We then tried to screen for peptides that may be involved in adipocyte thermogenesis regulation. Specifically, the secretion of BATSP1 was strikingly induced by FSK signaling (Fig. 1C); BATSP1 is composed of AA 160–178 of short-chain enoyl-CoA hydratase (ECHS1) and has the sequence FAQPEILIGTIPGAGGTQR (Fig. S2A). Multiple sequence alignment revealed that this domain is highly conserved between humans and mice (Fig. S2B). We next synthesized FITC-labeled BATSP1 and found that the peptide clearly entered both brown and white adipocytes and dispersed throughout the cytoplasm (Fig. 1D). These data indicate the physiological regulation of BATSP1 in adipose cells. BATSP1 is a bona fide regulator of adipose thermogenesis that was specifically screened from the upregulated secreted peptides. UCP1 protein expression was markedly induced by BATSP1 in brown adipocytes (Fig. 1E). The OCR was also increased by BATSP1, as indicated by measurements of the basal OCR, proton leakage, ATP production and maximal respiration (Fig. 1F and Fig. S2C). At the molecular level, the transcript levels of thermogenic genes (Ucp1, Pparα, Pgc1α, Cpt1α, Cideα and Cyt C) were also increased in BATSP1-treated brown adipocytes (Fig. 1G). To assess whether BATSP1 can potentiate thermogenesis in white adipocytes, fully differentiated human primary white adipocytes were treated with synthesized BATSP1. In concordance with the above results, the UCP1 expression level, the OCR and the levels of thermogenic markers were significantly elevated in response to BATSP1 (Fig. 1E , H, I and Fig. S2C). We finally performed a Cell Counting Kit-8 assay to exclude the possibility of cytotoxic effects of BATSP1, and we found that BATSP1 had no effect on cell viability (Fig. S2D). Together, these results strongly suggest that BATSP1 is a brown adipokine that is tightly linked to the activation of adipose thermogenesis.

### BATSP1 promotes adipose thermogenesis in mice

**Fig. 2:**
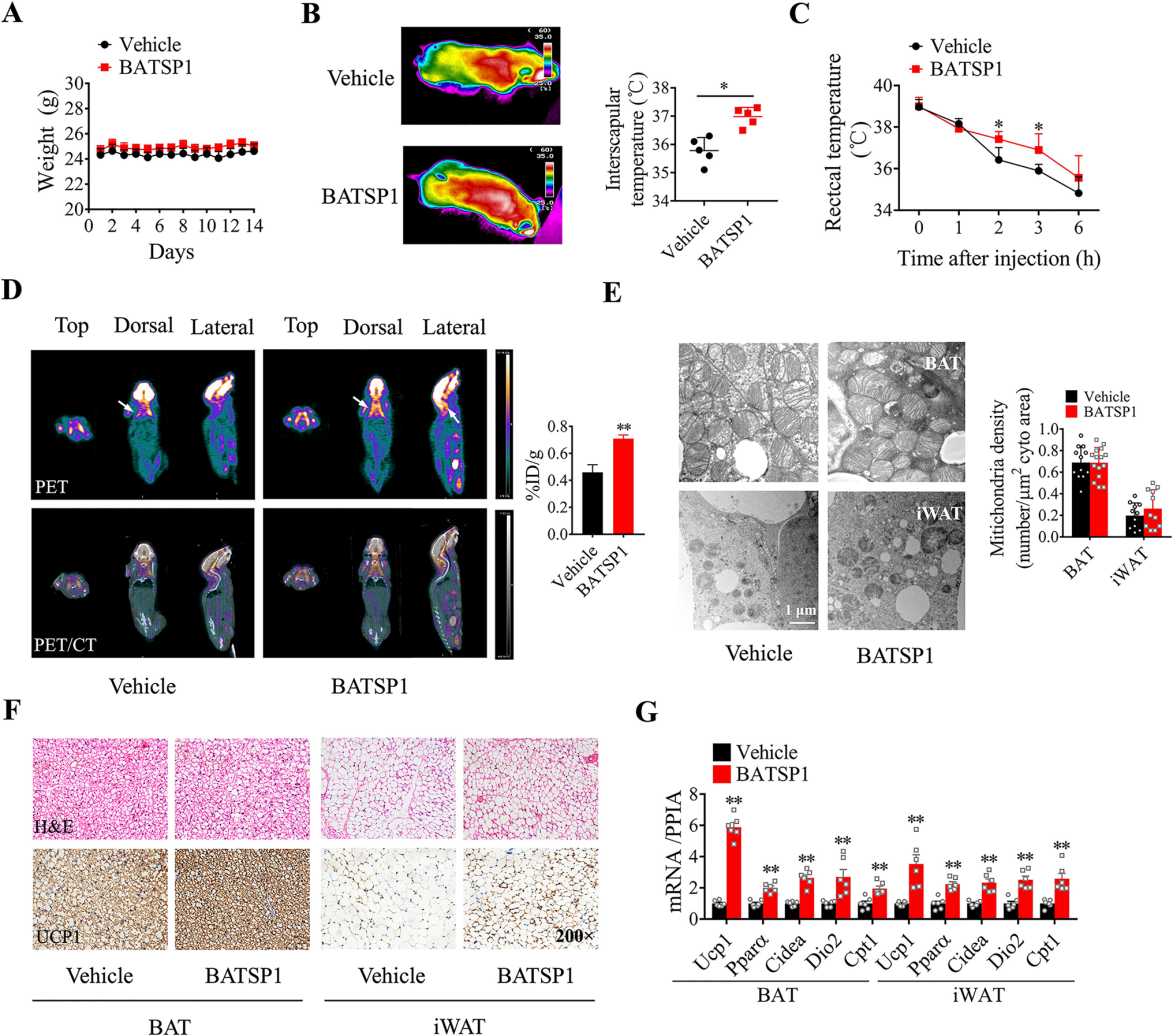
BATSP1 promotes adipose thermogenesis in mice. C57BL/6J mice fed a chow diet were injected intraperitoneally with 5 mg/kg/day BATSP1 or vehicle (saline) for 14 days (n = 5 mice/group). **A** Body weight time course. **B** Representative thermal images (left) and calculated interscapular temperatures (right). **C** Measurements of rectal temperatures in mice housed at 4 °C. **D** PET/CT images of mice after injection of ^18^F-FDG. **E** Representative transmission electron micrographs of BAT and iWAT. **F** Representative histological data (H&E and immunochemical staining of UCP1) of BAT and iWAT. **G** Expression of marker genes related to thermogenesis. The data are presented as the means ± SDs; *p < 0.05; **p < 0.01 by unpaired Student’s *t* test

We next revealed the tissue distribution of BATSP1 in mice by i.p. injection. The signal produced by FITC was higher in the liver, kidney, iWAT and eWAT—and to a lesser extent, in BAT and muscle—3 h after injection (Fig. S3A), suggesting that BATSP1 could be taken up by adipose tissues and perform its function. To further investigate the function of BATSP1 in vivo, C57BL/6 J mice fed a chow diet were treated with BATSP1 or vehicle control for 2 weeks. BATSP1 administration in vivo did not markedly affect body weight or food intake (Fig. 2A and Fig. S3B) or the serum concentrations of glucose and insulin under fasting conditions (Table S3). However, the surface temperature specifically at the interscapular region corresponding to the location of BAT was significantly increased after BATSP1 treatment (Fig. 2B), and the mice also exhibited marked resistance to cold (Fig. 2C). In addition, BATSP1-treated mice showed increased glucose uptake in BAT compared with vehicle control mice, as visualized by PET/CT imaging (Fig. 2D). However, no major difference was observed in mitochondrial biogenesis, as evaluated by TEM (Fig. 2E). Histological analysis revealed markedly increased expression of UCP1 in both BAT and iWAT; however, the volume of adipocytes was not affected by BATSP1 (Fig. 2F). The mRNA and protein expression of thermogenic genes was consistently induced by BATSP1 (Fig. 2G and Fig. S3C). These experiments suggest that BATSP1 is involved in regulating adipose thermogenesis in vivo, whereas these changes were attenuated when scrambled peptides were used (Fig. S3D–F). Furthermore, we observed no differences in the serum concentrations of alanine aminotransferase (ALT) and aspartate aminotransaminase (AST), biomarkers of liver damage (Table S3), between these two groups of mice. Additionally, no histological changes were observed in other tissues, such as brain, heart, kidney, liver, lung, spleen, intestine, pancreas and muscle tissue (Fig. S3G), indicating the lack of obvious side effects of BATSP1.

### BATSP1 increases whole-body energy expenditure in vivo

**Fig. 3:**
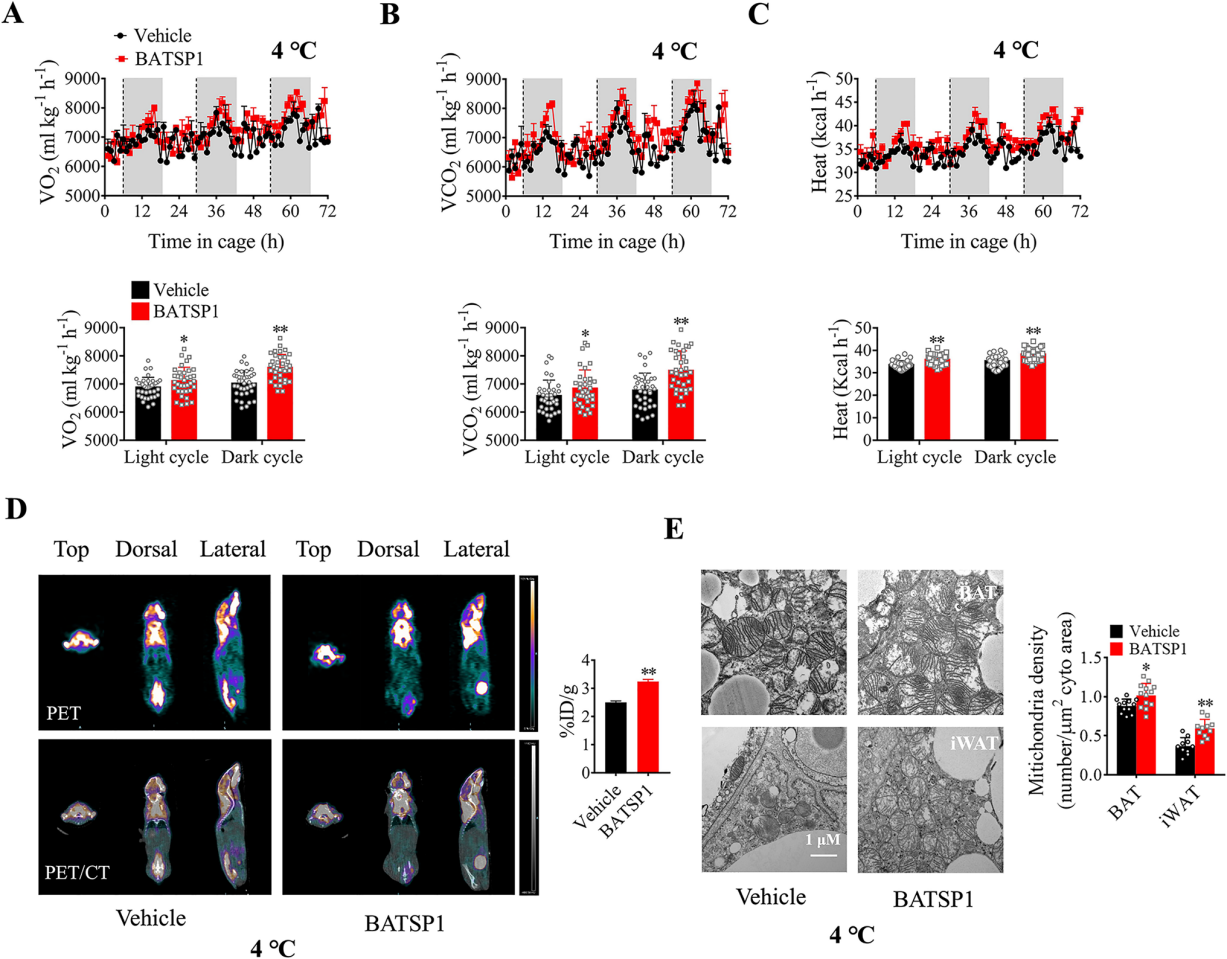
BATSP1 increases whole-body energy expenditure in vivo*.* Mice were treated with BATSP1 for 14 days and then exposed to 4 °C for 3 days (n = 5/group). Whole-body energy expenditure was evaluated by measuring oxygen consumption (*V*O_2_) (**A**), carbon dioxide release (*V*CO_2_) (**B**) and heat production (**C**). **D** ^18^F-FDG PET/CT imaging. **E** Representative electron micrographs of BAT and iWAT. The data are presented as the means ± SDs; *p < 0.05; **p < 0.01 by unpaired Student’s *t* test

To further investigate the effects of BATSP1 on whole-body metabolism, we performed metabolic cage studies with BATSP1-treated mice for 2 weeks. Interestingly, despite the tendency toward increased thermogenesis, whole-body energy expenditure under ambient conditions (25 °C) was not significantly different (Fig. S4A–E). We then asked whether thermogenically demanding conditions are required for the function of BATSP1. Mice were housed at 4 °C for 3 days after 2 weeks of BATSP1 injection. As shown in Fig. 3A–C, BATSP1 increased whole-body oxygen consumption (*V*O_2_) and CO_2_ production (*V*CO_2_) and elevated heat generation after cold exposure. However, the respiratory exchange ratio (RER) and locomotor activity were unchanged (Fig. S4F and G). We then confirmed that the increase in oxygen consumption was due to the upregulation of BAT activity in cold-exposed mice. Notably, the uptake of ^18^F-FDG, a glucose tracer, was significantly increased, as determined by PET/CT imaging (Fig. 3D). Accordingly, the mitochondrial content was substantially increased in mice injected with BATSP1 compared with those injected with vehicle control upon exposure to a cold environment (Fig. 3E).

### BATSP1 ameliorates diet-induced obesity under mild cold exposure

**Fig. 4:**
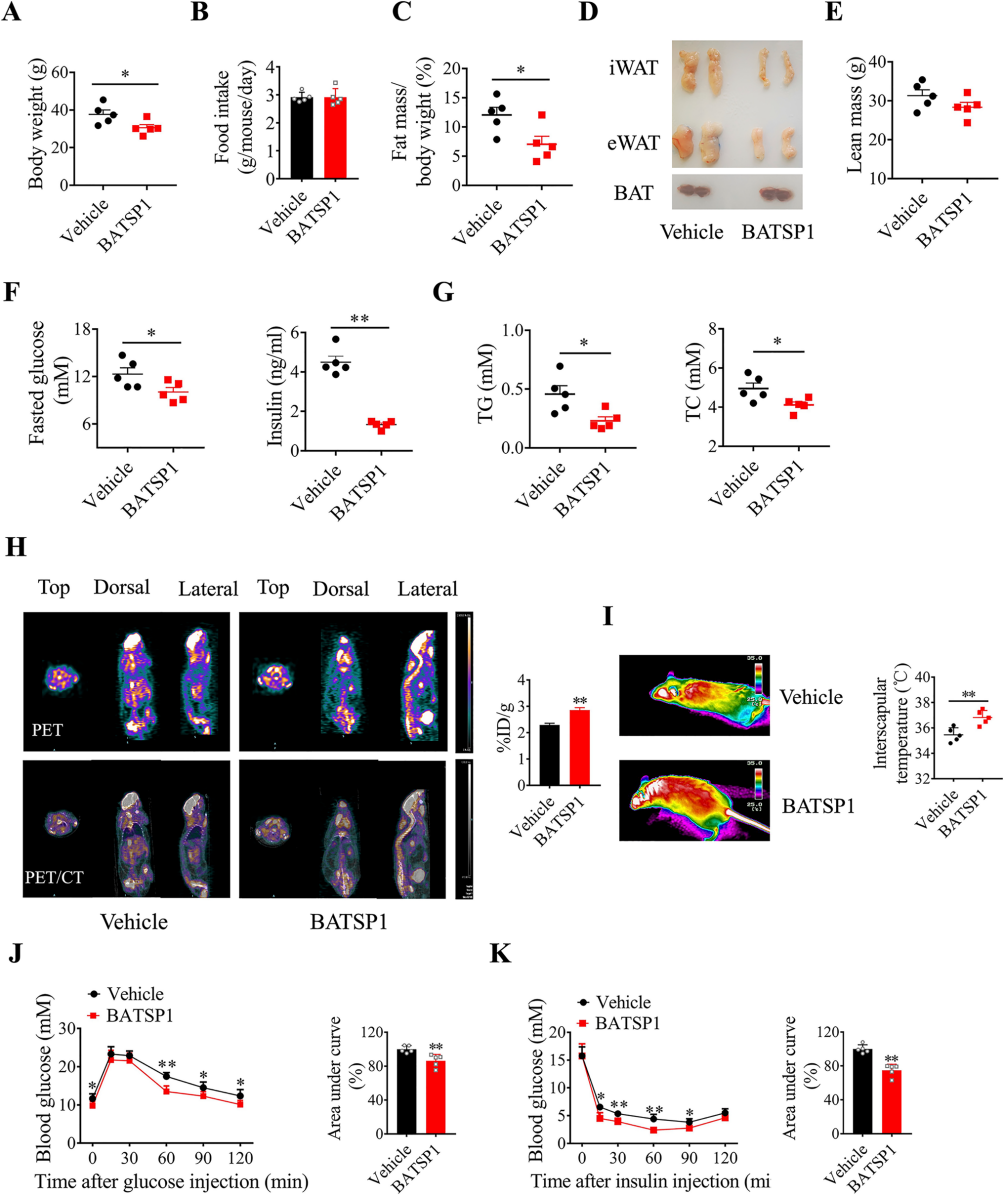
BATSP1 ameliorates diet-induced obesity under mild cold exposure. Mice fed a HFD were housed at 16 °C and injected with 5 mg/kg BATSP1 or vehicle twice per week for a period of 16 weeks (n = 5 mice/group). **A**–**E** Body weight (**A**), food intake (**B**), fat mass ratio (**C**), morphology of fat tissues (**D**) and lean mass (**E**) of HFD-fed mice. **F** Serum glucose and insulin concentrations. **G** Lipid concentrations. **H** ^18^F-FDG PET/CT imaging. **I** Representative thermal images (left) and calculated interscapular temperatures (right). **J**, **K** A GTT and an ITT were performed in HFD-fed mice treated prophylactically with BATSP1 or vehicle control. The data are presented as the means ± SDs; *p < 0.05; **p < 0.01 by unpaired Student’s *t* test

BATSP1 facilitates a negative energy balance by increasing adipose thermogenesis and therefore may hold promise for the development of novel anti-obesity approaches. To determine the potential therapeutic uses of BATSP1, mice fed a HFD were injected with BATSP1 twice a week. We first conducted the experiment with mice housed at room temperature. After 16 weeks of treatment, no significant reduction in body weight gain or food intake was observed in the BATSP1 group (Fig. S5A and B) compared with the control group (injected with vehicle). However, we did observe a higher skin temperature in the BAT region in BATSP1-treated mice fed a HFD (Fig. S5C). Consistent with this finding, the expression of UCP1 was strongly induced in BAT and iWAT, as indicated by histological and Western blot analyses (Fig. S5D and E). The effects of BATSP1 on energy expenditure may be masked at room temperature, at which no appreciable cold stimulation occurs and heat production is only partially activated. Therefore, we conducted a second experiment to determine whether the increased thermogenesis induced by chronic BATSP1 injection leads to a reduction in the mass of mice upon cold stress. Notably, mice housed in a mildly cold environment (16 °C) exhibited marked resistance to HFD-induced body weight gain (Fig. 4A), although their food intake was similar to that of control mice (Fig. 4B). Lipid stores were accordingly decreased in iWAT and eWAT pads after 16 weeks of treatment, with no effect on lean mass (Fig. 4C–E). BATSP1 also induced pronounced decreases in fasting serum glucose and insulin (Fig. 4F), as well as in the concentrations of triglycerides (TG) and total cholesterol (TC) (Fig. 4G).

To determine whether this body weight loss results from the stimulation of BAT thermogenesis, we evaluated the phenotypic effects of BATSP1 treatment on BAT and iWAT. ^18^F-FDG PET/CT scanning showed that glucose uptake was robustly increased in BATSP1-treated mice in the cold environment (Fig. 4H). Consistent with this finding, the surface temperature was significantly increased in the interscapular region by BATSP1 treatment (Fig. 4I). Hematoxylin and eosin (H&E) staining revealed that BAT and iWAT from BATSP1-treated mice contained smaller lipid droplets (Fig. S5F). Immunohistological staining indicated that BATSP1 treatment in a cold environment resulted in an increase in the UCP1 protein level (Fig. S5F). Consistent with this result, Western blot analysis showed 1.7-fold and 1.6-fold increases in UCP1 expression in BAT and iWAT, respectively, in BATSP1-treated animals (Fig. S5G). Along with a decreased body weight and reduced fat mass, BATSP1-treated mice also exhibited marked improvements in glucose handling and insulin action (Fig. 4J, K), as revealed by the GTT and ITT. These data demonstrate a new function for BATSP1 in mediating resistance to HFD-induced obesity and improving certain aspects of metabolic health under cold exposure.

### BATSP1 regulates the subcellular localization of FOXO1

To delineate the molecular mechanism responsible for BATSP1-regulated thermogenesis, we performed RNA sequencing (RNA-seq) in BATSP1-treated brown adipocytes. Transcripts with a fold change of ≥ 2 and false discovery rate-adjusted p value (q value) of < 0.05 were considered differentially expressed genes. A total of 89 genes with significant differences in expression were identified (Table S4), of which 27 were upregulated and 62 were downregulated (Fig. S6A and B). Pathways associated with ABC transporters, pathogenic *Escherichia coli* infection and the FoxO signaling pathway were enriched in these genes (Fig. S6C). Specifically, FOXO1, a key player in the FOXO family, plays a crucial role in adipocyte metabolism as a common transcription factor [15]. To directly test the involvement of the FOXO1 signaling pathway, we first determined the expression level of FOXO1. However, BATSP1 treatment did not alter the mRNA level of FoxO1 in either brown or white adipocytes (Fig. S6D). Consistent with this finding, the protein expression level of FOXO1 was also unaffected in these cells upon BATSP1 treatment (Fig. S6E). Thus, the transcription and translation of FOXO1 cannot mechanistically explain the thermogenic reprogramming mediated by BATSP1.

**Fig. 5:**
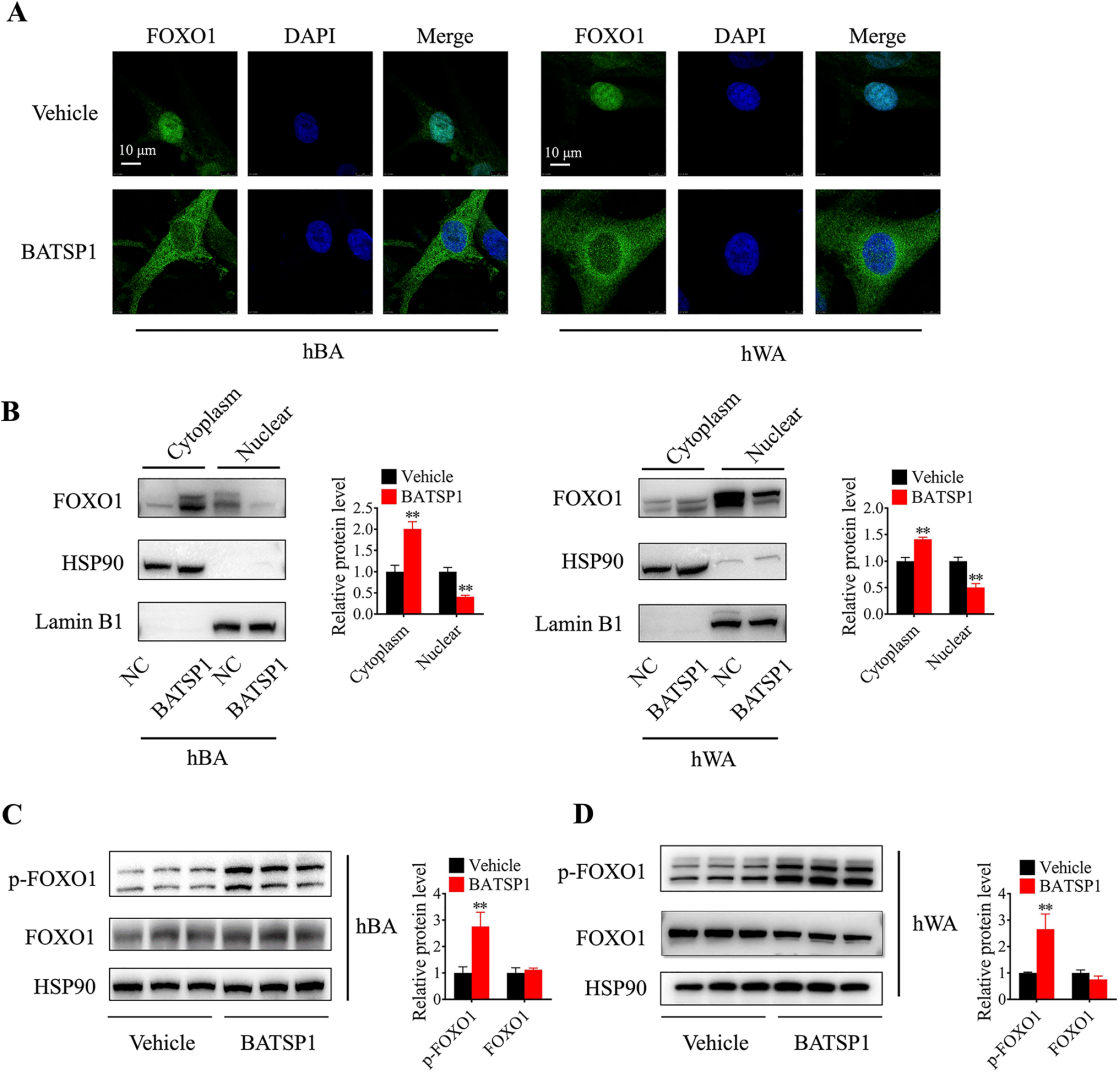
BATSP1 regulates the subcellular localization of FOXO1. **A** Subcellular localization of FOXO1 in brown and white adipocytes treated with or without BATSP1 for 3 h. **B** Human brown and white adipocytes were treated as indicated in Panel **A**, and the cytosolic and nuclear fractions were isolated and analyzed by Western blotting. HSP90 and Lamin B1 were used as controls for the cytosolic and nuclear fractions, respectively. **C**, **D** Representative immunoblot showing the level of FoxO1 phosphorylated at S256 in brown and white adipocytes treated with or without BATSP1. The data are presented as the means ± SDs; **p < 0.01 by unpaired Student’s *t* test

Accumulating studies have highlighted the physiological role of nucleocytoplasmic shuttling of FOXO1 in regulating its activity [15], thus affecting UCP1 transcription. To precisely visualize the subcellular localization of FOXO1 in response to BATSP1 stimuli, we performed immunofluorescence staining in primary brown and white preadipocytes. As shown in Fig. 5A, FOXO1 remained in the nucleus under normal conditions in vehicle-treated cells, while BATSP1 stimulation decreased the content of FOXO1 in the nucleus and increased cytosolic FOXO1 accumulation, indicating that BATSP1 is involved in FOXO1 nuclear exclusion. Western blot analysis of subcellular fractions isolated from BATSP1-treated brown and white adipocytes showed similar trends (Fig. 5B). Since phosphorylation has been shown to be responsible for FOXO1 nuclear exclusion, we analyzed FOXO1 phosphorylation at S256, which directly affects its translocation. As expected, the level of phosphorylated FOXO1 was significantly elevated by BATSP1 in adipocytes (Fig. 5C, D). Therefore, BATSP1 controls the subcellular localization of FOXO1 and simultaneously affects its transcriptional function.

### BATSP1 releases the transcriptional inhibition of UCP1 by FOXO1

**Fig. 6:**
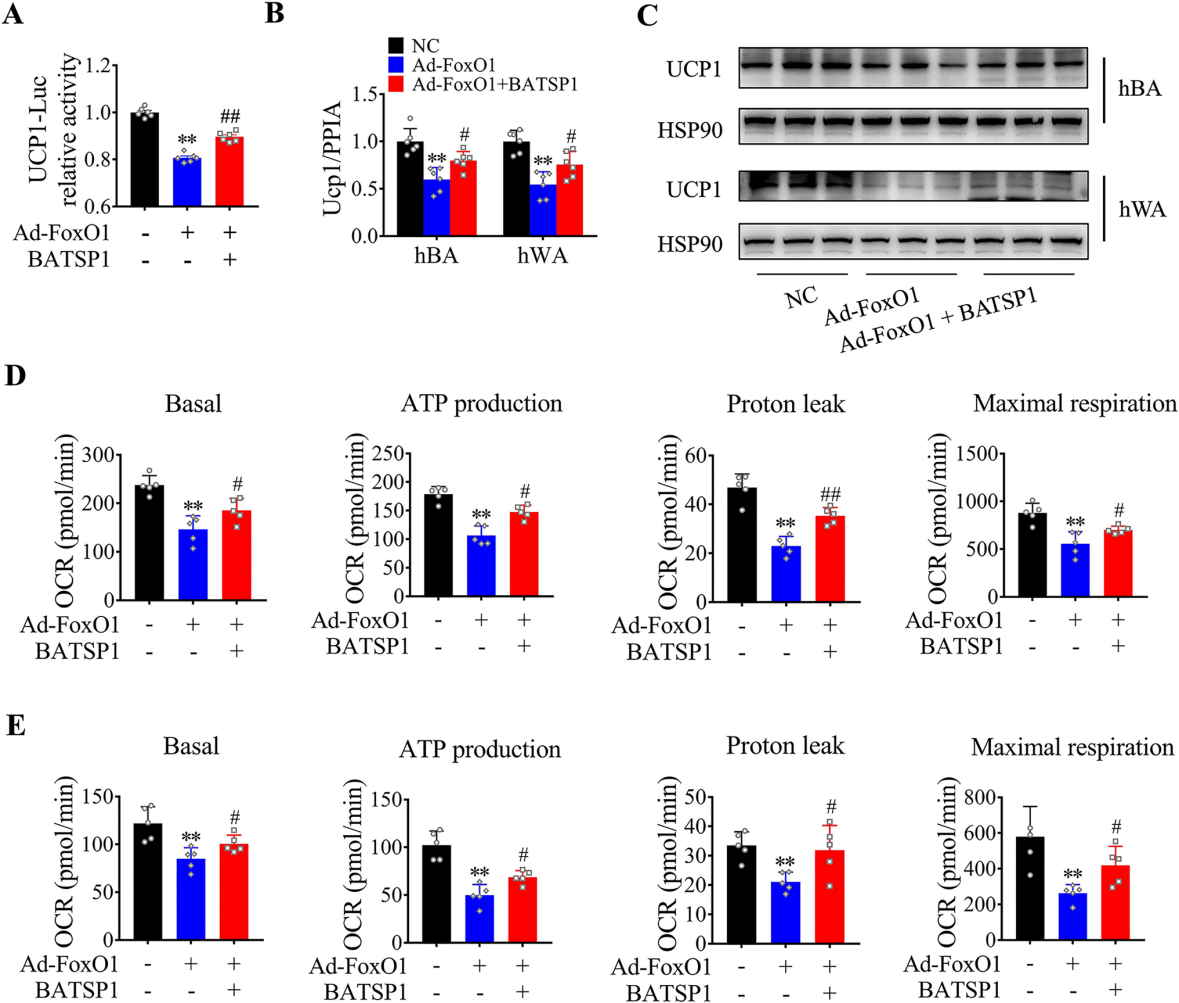
BATSP1 releases the transcriptional inhibition of UCP1 by FOXO1. **A** Reporter assays in HEK293T cells infected with adenoviruses expressing FOXO1 or mock viruses and treated with BATSP1 or vehicle control. **B**, **C** RT‒qPCR and Western blot analyses of UCP1 expression in vitro. Brown and white adipocytes were transduced with control or FOXO1-encoding adenoviral vectors, and differentiation was then initiated prior to stimulation with BATSP1 or treatment with vehicle control for 6 h. **D**, **E** Continuous measurement of oxygen consumption in adipocytes isolated from BAT and treated as described in Panels **B** and **C**. The data are presented as the means ± SDs; **p < 0.01; ^#^p < 0.05; ^##^p < 0.01 by unpaired Student’s *t* test

Because FOXO1 has been shown to inhibit UCP1 gene expression [16], we analyzed the effect of BATSP1 on UCP1 transcriptional activity. We then used a dual human Ucp1-luciferase reporter system to examine the role of FOXO1 translocation resulting from BATSP1 treatment in controlling Ucp1 gene transcription. As shown in Fig. 6A, the luciferase reporter assay showed that FoxO1 overexpression mediated by adenoviral transduction indeed suppressed Ucp1 transcription, whereas BATSP1 stimulation partially restored Ucp1 transcription, increasing it by 45%. In accordance with the released inhibition of UCP1 transcription after BATSP1 treatment, we observed increased UCP1 expression in brown and white adipocytes (Fig. 6B, C and Fig. S6F). To further confirm the importance of BATSP1-FOXO1 signaling in regulating adipose thermogenesis in vitro, we measured cellular respiration with a Seahorse extracellular flux analyzer. Our findings showed that BATSP1 antagonized the effect of FOXO1 and decreased oxygen consumption in both brown and white adipocytes, as indicated by measurements of the basal OCR, ATP production, proton leakage and maximal respiration (Fig. 6D, E and Fig. S6G). Thus, BATSP1-FOXO1 signaling is implicated in regulating thermogenesis in adipocytes through control of the subcellular localization of FOXO1 and restoration of Ucp1 expression.

Previous studies indicate that FOXO-binding proteins, including Zfp38, 14–3-3 Z and FCoR [17–19], are implicated in FOXO1-mediated transcriptional repression. We thus measured the expression levels of these potential targets in brown and white adipocytes upon BATSP1 treatment. First, we measured the transcript levels of these genes in adipocytes by RT‒qPCR analysis and found that they were not influenced by BATSP1 (Fig. S7A), suggesting that the regulation of BATSP1 does not occur at the transcriptional level. Second, we compared the protein levels of these genes in adipocytes treated with BATSP1. However, the protein expression levels of ZFP238 and 14-3-3 Z were comparable in BATSP1-treated and control adipocytes (Fig. S7B). Regrettably, no antibodies against FCoR are commercially available. Based on these findings, we conclude that these genes may not be involved in FOXO1-mediated repression of UCP1 transcription after BATSP1 stimulation. The target of BATSP1 that regulates the thermogenic program in cooperation with Foxo1 remains to be further clarified.

## Discussion

The involvement and nature of interorgan crosstalk in regulating systemic energy homeostasis, especially the secretory role of BAT, is a new frontier in obesity therapeutics. However, the repertoire of secreted factors released by brown adipocytes and the potential beneficial effects of these factors on target organs have not been fully defined. Here, we provide a comprehensive profiling analysis of secreted peptides released from human brown adipocytes under basal conditions and acute FSK stimulation. Numerous novel extracellular peptides secreted from human brown adipocytes in response to thermogenic stimuli were identified. Specifically, we focused on the role of BAPSP1, which promotes thermogenesis in brown and white adipocytes. When administered at a pharmacological concentration in vivo, BAPSP1 can robustly activate thermogenesis in BAT and induce WAT browning, consequently leading to resistance to diet-induced obesity. Mechanistically, BAPSP1 increases the nuclear exclusion of FOXO1, which is followed by the release of Ucp1 transcriptional repression to allow it to perform its functions.

In recent years, extensive efforts have been made to recognize the secretory role of BAT; these findings have helped to expand the functional importance of BAT in whole-body energy regulation. The effects of some of these molecules, or batokines, are mediated through an autocrine mechanism that locally modulates BAT development and thermogenesis [3, 4]. For example, Slit2-C, the C-terminal fragment of Slit2, has been reported to positively affect adipose thermogenesis, thus increasing energy expenditure and improving glucose homeostasis in vivo [20]. Wang YX and his collaboration identified a novel BAT-enriched adipokine, Adissp, which exerts both paracrine and endocrine effects to promote WAT thermogenesis [21]. However, tremendous efforts are still needed to obtain a comprehensive understanding of the actors and actions involved in the endocrine properties of BAT. Technological innovations have made peptidomic analysis a useful tool for analyzing the secretory function of BAT [12]. Therefore, we performed peptidomic analysis of human brown adipocytes to obtain thorough knowledge of peptide factors secreted upon FSK treatment. Our results reinforce the idea that thermogenic stimuli led to distinct secretory profiles and thus provide insights into molecular mechanisms that may influence metabolic functions. BAPST is a peptide secreted by human brown adipocytes that has one of the greatest increases in abundance in response to thermogenic signaling. The positive effects of BAPST on promoting BAT thermogenesis and inducing WAT browning suggest that it functions in both autocrine and paracrine manners.

FOXO1 is a central regulator of metabolism in adipocytes, mediating key events in glucose homeostasis, lipolysis and differentiation [22–24]. Importantly, FOXO1 has been shown to repress the transcription of UCP1, and this inhibitory effect of FOXO1 is mainly mediated by Akt-induced phosphorylation of FOXO, which promotes the shuttling of FOXO from the nucleus to the cytoplasm [15, 16]. Our RNA-seq results revealed that BATSP1 promotes FOXO1 signaling. We further revealed that this effect is mediated via regulation of FOXO1 phosphorylation, leading to FOXO1 nuclear exclusion and releasing UCP1 from transcriptional repression. Interestingly, the function of BATSP1 in protecting against HFD-induced obesity was manifested only under mildly cold conditions and not at ambient temperature. We interpreted that cold stress has an additive effect on facilitating FOXO1 shuttling. Thus, BATSP1 may further ensure a full thermogenic response in adipose tissue. On the other hand, we could not exclude the possibility that FOXO1 is not the only target of BATSP1; other BATSP1 target genes may also have functional roles related to obesity.

Endogenous peptides are naturally occurring peptides that are widely present in tissues/cells, body fluids, exosomes, etc. [25, 26]. With the in-depth understanding of the production of endogenous peptides, diverse sources have been discovered and characterized. Specifically, one typical source of endogenous peptides is genetic encoding by coding or noncoding genes, resulting in the generation of peptides such as short open reading frame (sORF)-encoded peptides [26]. For example, MOTS-c, which is transcribed from mitochondrial DNA, shows antiobesity effects by activating the AMPK pathway [27]. The majority of endogenous peptides are produced during protein degradation, which involves multiple steps and processes such as ubiquitin‒proteasome-mediated degradation, enzymatic degradation, or degradation through other pathways [28]. Examples of bioactive peptides generated through precursor degradation include hemopressin peptides derived from fragments of alpha-hemoglobin, and erythropoietin (EPO)-derived Helix B-surface peptide (pHBSP) [29, 30]. Although historically misunderstood as junk products of protein degradation, fragment peptides are currently realized to play key roles in various cell functions. Here, we demonstrated that BATSP1, derived from AA 160–178 of ECHS1, plays important roles in adipose thermogenesis. This finding might provide a novel therapeutic implication for obesity prevention. However, it is regrettable that the enzymes that generate and degrade BATSP1 and the mechanisms by which these processes are regulated remain unclear.

## Conclusions

Our study provided a comprehensive overview of peptides secreted from human brown adipocytes upon exposure to thermogenic stimuli. Specifically, we focused our study on BATSP1 and confirmed its effects on energy expenditure and body weight loss, and our findings suggest the future potential application of BATSP1 in the treatment of metabolic disorders. However, there are still some limitations in our present study. For example, the mechanism by which BATSP1 increases adipose thermogenesis is still not entirely understood, and we failed to identify a direct target of BATSP1. Additionally, most experiments in the present study were performed in mouse models; however, clinical studies will help to fully assess the therapeutic potential of BATSP1. Mechanistically, we could not exclude the possibility that BATSP1 may act through a receptor-mediated mechanism, although it predominantly enters adipocytes. Finally, the concentration of BATSP1 is high, and future efforts are needed to improve the pharmacokinetic and pharmacodynamic properties of BATSP1 to treat metabolic disorders.

### Supplementary Information

Below is the link to the electronic supplementary material.

Supplementary file1 (XLSX 661 KB)

Supplementary file2 (XLSX 1084 KB)

Supplementary file3 (XLSX 10 KB)

Supplementary file4 (XLS 56 KB)

Supplementary file5 (XLSX 12 KB)

Supplementary file6 (DOCX 10065 KB)

## Data Availability

All data associated with this study are present in the paper or the Supplementary Materials.
