# Supplementary material for: The secreted peptide BATSP1 promotes thermogenesis in adipocytes
Source: Cell Mol Life Sci. 2023 Nov 27;80(12):377. doi: 10.1007/s00018-023-05027-9 (PMC10682272; doi:10.1007/s00018-023-05027-9)
Supplement: Supplementary file 6 — Supplementary file6 (DOCX 10065 KB) [file 18_2023_5027_MOESM6_ESM.docx]

**Supplementary Materials for**

**The Secreted Peptide BATSP1 Promotes Thermogenesis in Adipocytes**

Xianwei Cui *et al.*

**Corresponding authors:** Chenbo Ji, chenboji@njmu.edu.cn.

**Supplementary Materials and Methods**

**Peptidomic analysis**

For peptidomics analysis, fully differentiated cells were washed with PBS and subsequently maintained in DMEM/F12 containing 1% penicillin-streptomycin for 2 h and then stimulated with 10 μM FSK (Sigma-Aldrich) for another 4 h. Cell supernatant was collected and passed through 0.45 μm filter to remove any floating cells, the filtrates were then centrifuged at 10000 × g at 4 ℃ to remove cell debris. 20% acetonitrile was added to the supernatants to disrupt the protein–peptide interactions. The mixture was centrifuged through the 10 KDa cut-off filter to extract the peptides. The flow-through was dried by a vacuum-dried by a Labconco’s freeze dryer. Powders were redissolved in 0.1% formic acid and subsequently analyzed by LC–MS/ MS.

The MS analysis was performed following the below description. The peptides mixture was firstly labeled with TMT reagents according to the manufacturer’s instruction (Thermo Fisher Scientific), and then separated with an LC packings C18 column (Acclaim PepMap, 75 μm × 150 mm) coupled to an Ultimate 3000 nano‐LC system (Eksigent Technologies, Dublin, CA). The obtained elutions were directly injected into an LTQ-Orbitrap Velos mass spectrometer (Thermo Fisher Scientific, USA). The MS data were collected and searched using the Mascot database search (http://www.matrixscience.com) against the SwissProt sequence database (<http://www.expasy.org/> tools/) considering the following variable modifications: phosphorylation, carbamylation, deamidation, methionine oxidation, acetylation, etc. PEAKS software (version 7.0, Bioinformatics Solutions) was also performed to search the databases using MS/MS spectral data.

**Gene expression and immunoblotting analyses**

Total RNA was extracted from cultured cells or adipose tissue using TRIzol reagent (Invitrogen), and reverse transcription was performed with HiScript II 1st Strand cDNA Synthesis Kit (Vazyme Biotech, China). The relative gene expression was detected by RT-qPCR with SYBR green Master Mix (Thermo Fisher Scientific). The sequence of primers is listed in Table S5. Results were calculated using the comparative delta-delta-Ct method and were normalized to peptidylprolyl isomerase A (PPIA). For RNA-seq study, total RNA from human brown adipocytes receiving BATSP1 treatment was sequenced on the Illumina HiSeq X-ten (Illumina, USA). Data analyses and processing were performed according to the manufacturer’s instructions.

For Western blot analysis, total proteins of cells or adipose tissues were prepared in RIPA buffer. Protein lysates were separated on SDS–PAGE gels, transferred to PVDF membrane and immunoblotted with the following primary antibodies: anti-UCP1 (for immunoblotting in human samples) (ab155117, Abcam), anti-UCP1 (for immunoblotting in mouse samples) (23763-1-AP, Proteintech), anti-FOXO1 (2880, CST), anti-phospho FOXO1 Ser256 (9461, CST), anti-ZFP238 (12714-1-AP, Proteintech) and anti-14-3-3 Z (ab125032, Abcam). The HSP90 (Santa Cruz) was used as a loading control. Fluorescent images were captured by the ChemiDoc MP imaging system (Bio-rad, USA). Intensity values of bands were analyzed by the NIH Image J software (version 1.52i).

**Immunofluorescence staining and histology**

Human brown and white preadipocytes were plated in 35 mm confocal dishes and induced to fully differentiation. To study the cellular distribution, BATSP1 in conjunction with FITC was added to the medium and cultured for 2 h; then, cells were gently washed with PBS and stained with LipidTox Red 1:400 (Invitrogen) 10 min prior to imaging. To visualize the nuclear-cytoplasmic translocation of FOXO1, BATSP1-treated adipocytes were fixed with 4% paraformaldehyde and permeabilized with 0.5% Triton X-100. Cells were subsequently dyed with anti-FOXO1 antibody overnight at 4 ℃, and then incubated with Alexa Fluor 488-conjugated anti-rabbit secondary antibody (Jackson ImmunoResearch) for 1 h at room temperature. Finally, the cells were labeled by DAPI, and pictures were captured by a Stellaris 8 confocal microscope (Leica, German).

Tissues were fixed with 10% formalin, dehydrated and embedded in paraffin. For H&E staining, sections were cut and stained for H&E (Sigma-Aldrich) according to standard procedures. For immunochemical staining, adipose sections were deparaffinized and underwent rehydration and antigen retrieval in 1 × Antigen Retrieval Buffer (Abcam). Slides were blocked in 5% normal donkey serum in 1 × PBS and then incubated with primary antibody against UCP1 (23763-1-AP, Proteintech, 1:300) in 0.1% Triton X-100/ 1% BSA (Sigma-Aldrich) in a humidified chamber overnight at 4 ℃. Next day the horseradish peroxidase tagged secondary antibody was used to visualize the bonded antibody in combination with a commercially available DAB kit (Vector Laboratories). Hematoxylin was used for counterstaining. All pictures were acquired with a fluorescence microscope (Zeiss, Imager. A2, Germany).

**Cell viability**

Cell viability was evaluated using the Cell Counting Kit-8 (Beyotime, China). Briefly, human primary brown and white adipocytes were seeded in 96-well plates, and BATSP1 was introduced to cells with a concentration of 50 μM in culture medium. After 72 h, CCK-8 working solution was added into each well and incubated at 37 °C for an additional 3 h. Finally, the absorbance at 450 nm was measured using fluorescence multimode plate reader (Synergy 4, BioTek Instruments, Inc., USA).

**Blood and liver chemistry**

Blood was collected at the time when the mice were killed. Serum insulin levels were measured using the Rat/Mouse Insulin ELISA kit (Millipore # EZRMI-13K). Changes in the levels of TG and TC were determined using the colorimetric enzymatic method with commercial kits (Applygen Technologies Inc., China). Biomarkers of liver injury including ALT and AST were analyzed biochemically using related commercial kits (Abcam, USA). All operations were performed according to the protocol provided by the manufacturer.

**Supplemental Figures**

**
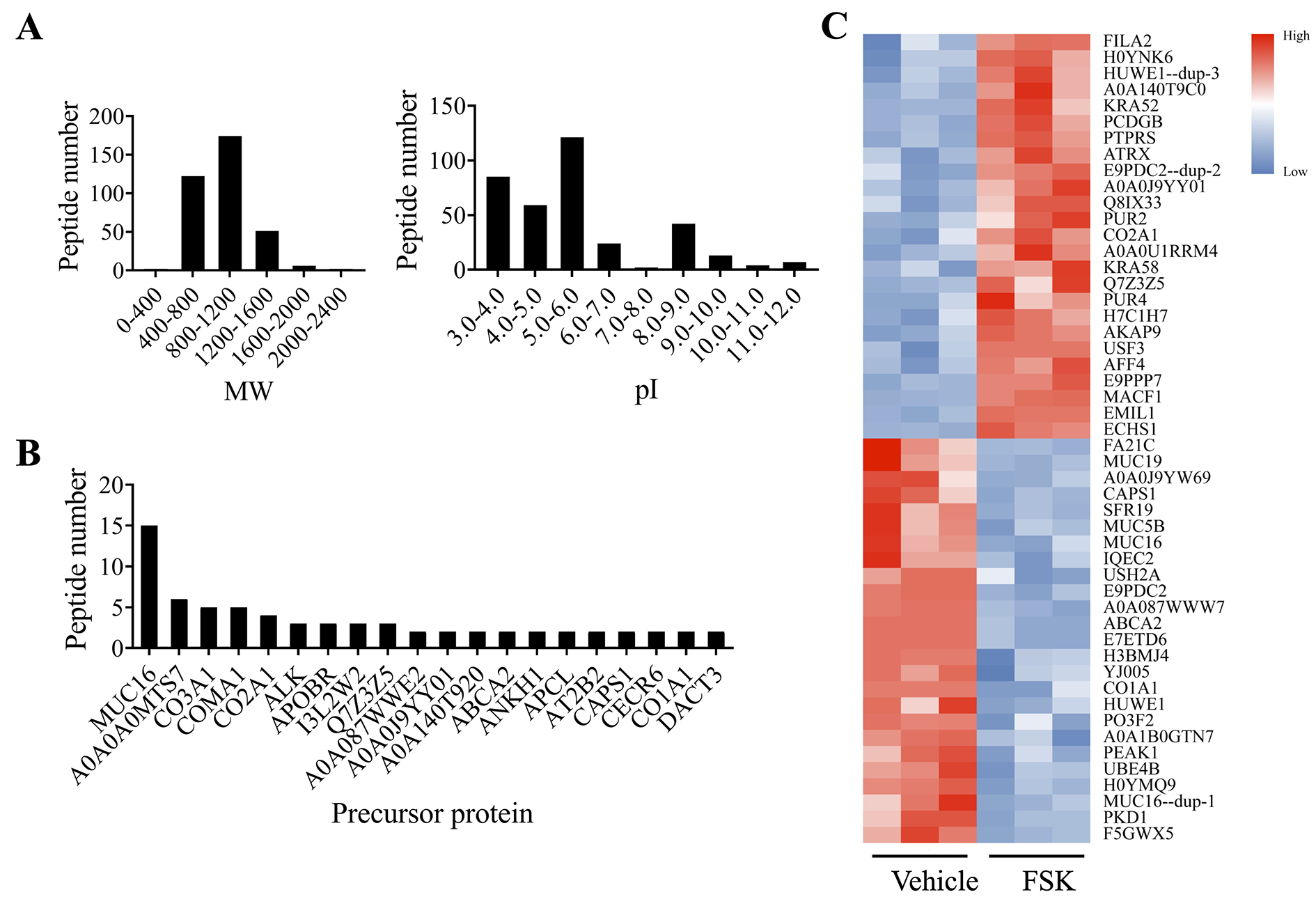
**

**Fig. S1 Characteristics of differentially expressed peptides in response to FSK**

(A) The molecular weight (MW) and isoelectric point (pI) distribution of identified peptides. (B) The number of peptides derived from the corresponding precursors. (C) Heat map showing the top 25 differentially expressed peptides.

**
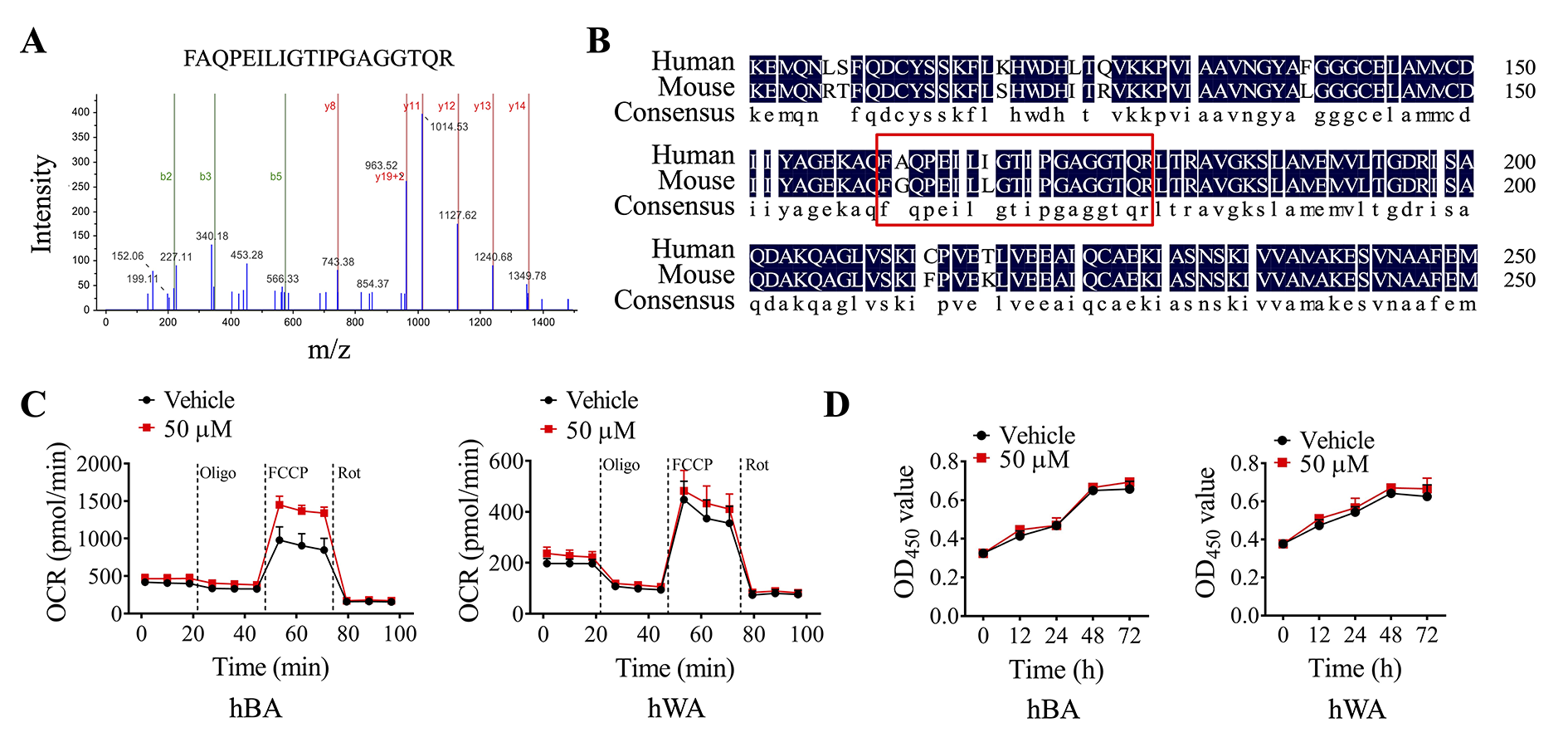
**

**Fig. S2 Identification of BATSP1 as a regulator of adipocytes thermogenesis**

(A) BATSP1-matched sequence identified by mass spectrometry. (B) Amino acid sequences alignment of BATSP1 between human and mouse. (C) Traces for the Seahorse experiments in human brown adipocytes (hBA) and white adipocytes (hWA). (D) Cell viability was determined by CCK-8.

**
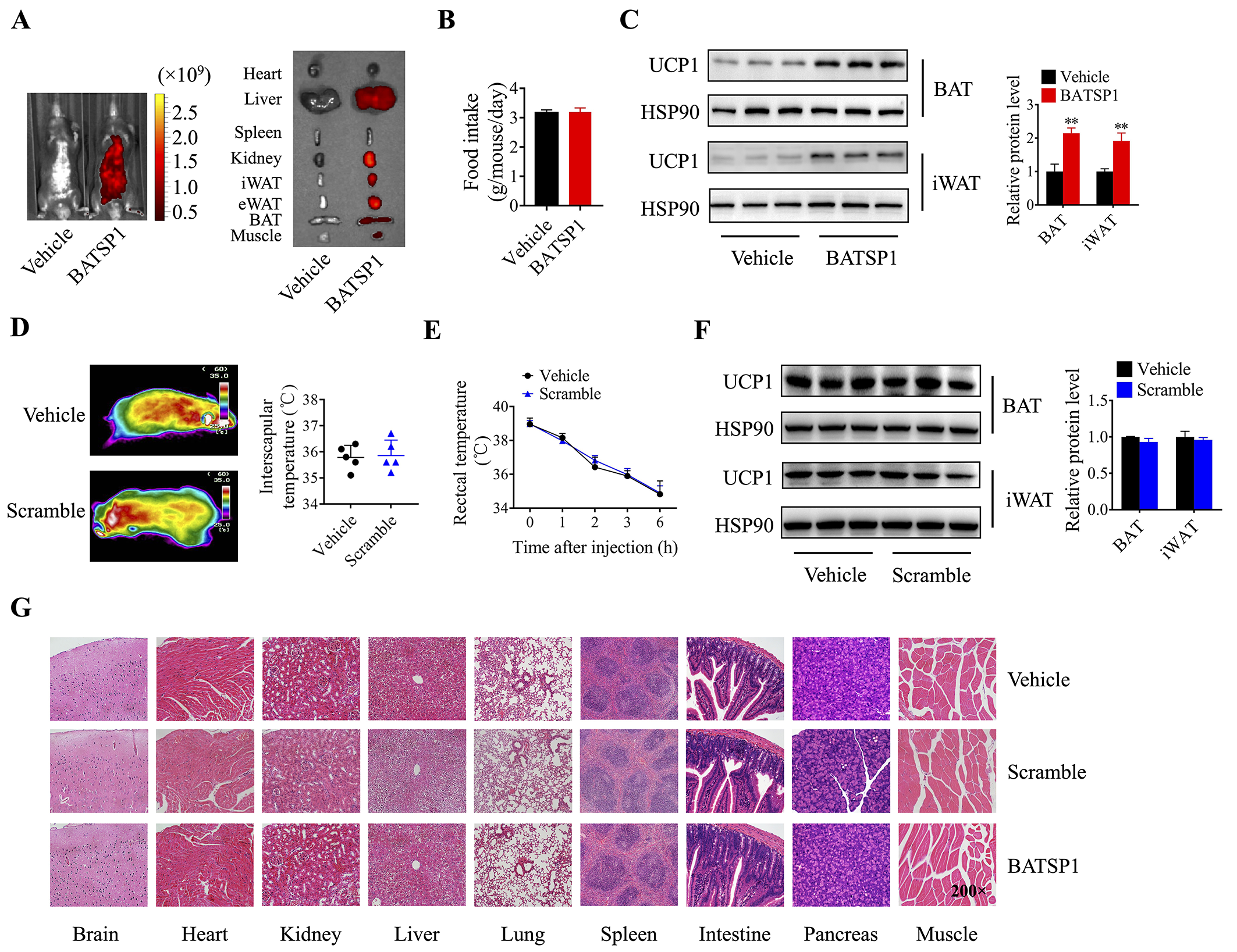
**

**Fig. S3 Scramble peptide causes no detectable changes in adipose thermogenesis**

(A) Tissue distribution of FITC-labeled BATSP1. (B) Food intake between BATSP1 and vehicle treatment groups. (C) Expressions of UCP1 were determined by Western blot after BATSP1 treatment. For D-F, mice were treated with scramble peptide (PEFIAGLPGGTGRTQIAQI) or vehicle for 14 days, delivered via i.p. injection (n = 5/group). (D) Representative infrared images (left) and surface temperature of the interscapular region (right). (E) Rectal temperature in scramble peptide- and vehicle-treated mice. (F) Immunoblot analysis of UCP1 in BAT and iWAT. (G) H&E staining of tissue sections. Data are mean ± SD; **, p < 0.01 by unpaired student *t*-test.

**
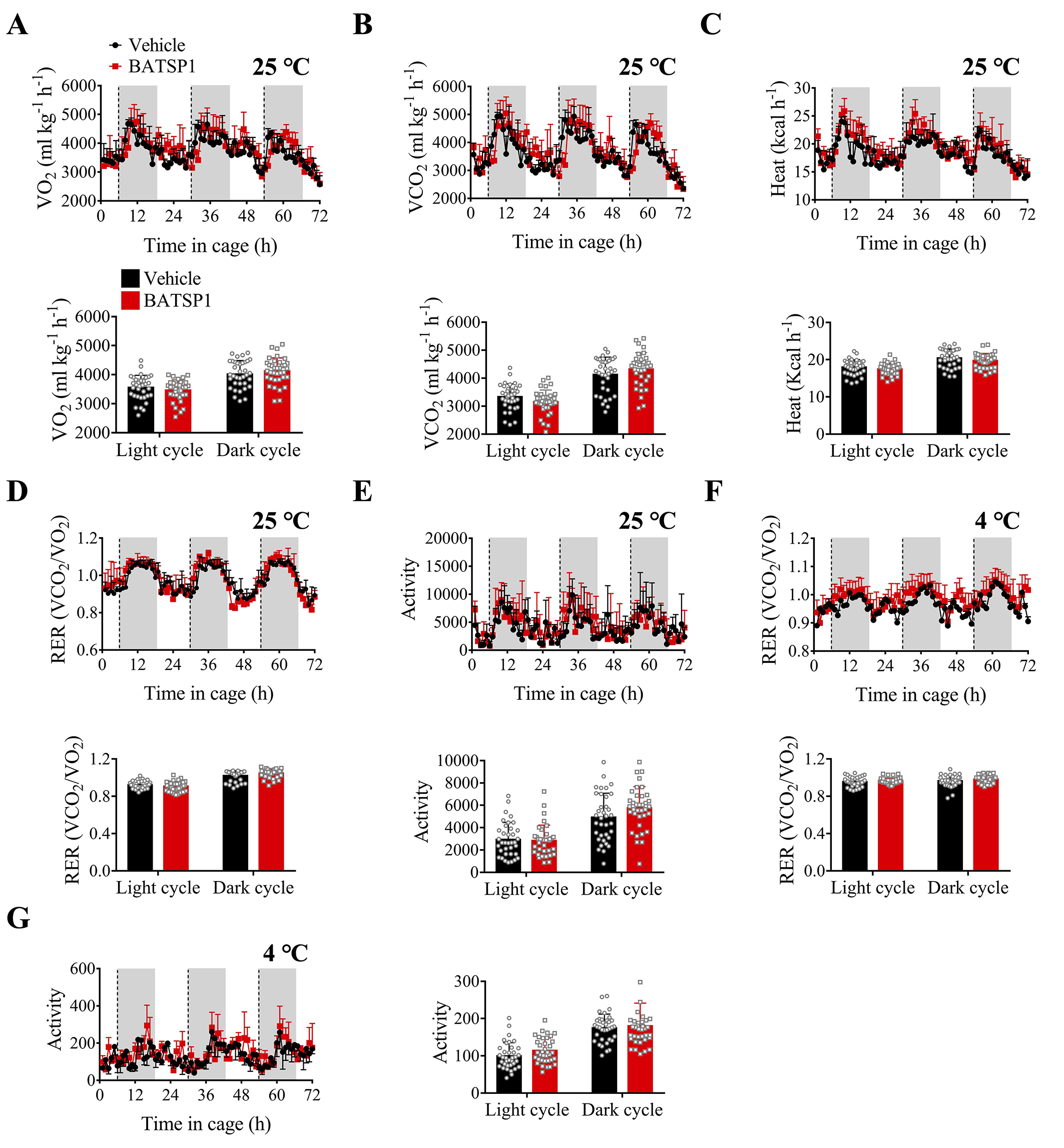
**

**Fig. S4 BATSP1 doesn’t increase energy expenditure at room temperature**

(A-E) O_2_ consumption (A), CO_2_ production (B), heat generation (C), RER (D) and locomotor activity (E) were measured in metabolic cages between BATSP1- and vehicle-treated mice housed at 25 °C (n=4/group). (F and G) Cold exposure at 4 °C for 3 days didn’t cause any difference in RER and locomotor activity in mice following 2 weeks of BATSP1 injection. Statistical analysis in dark and light phases is indicated at the bottom. Data are mean ± SD.

**
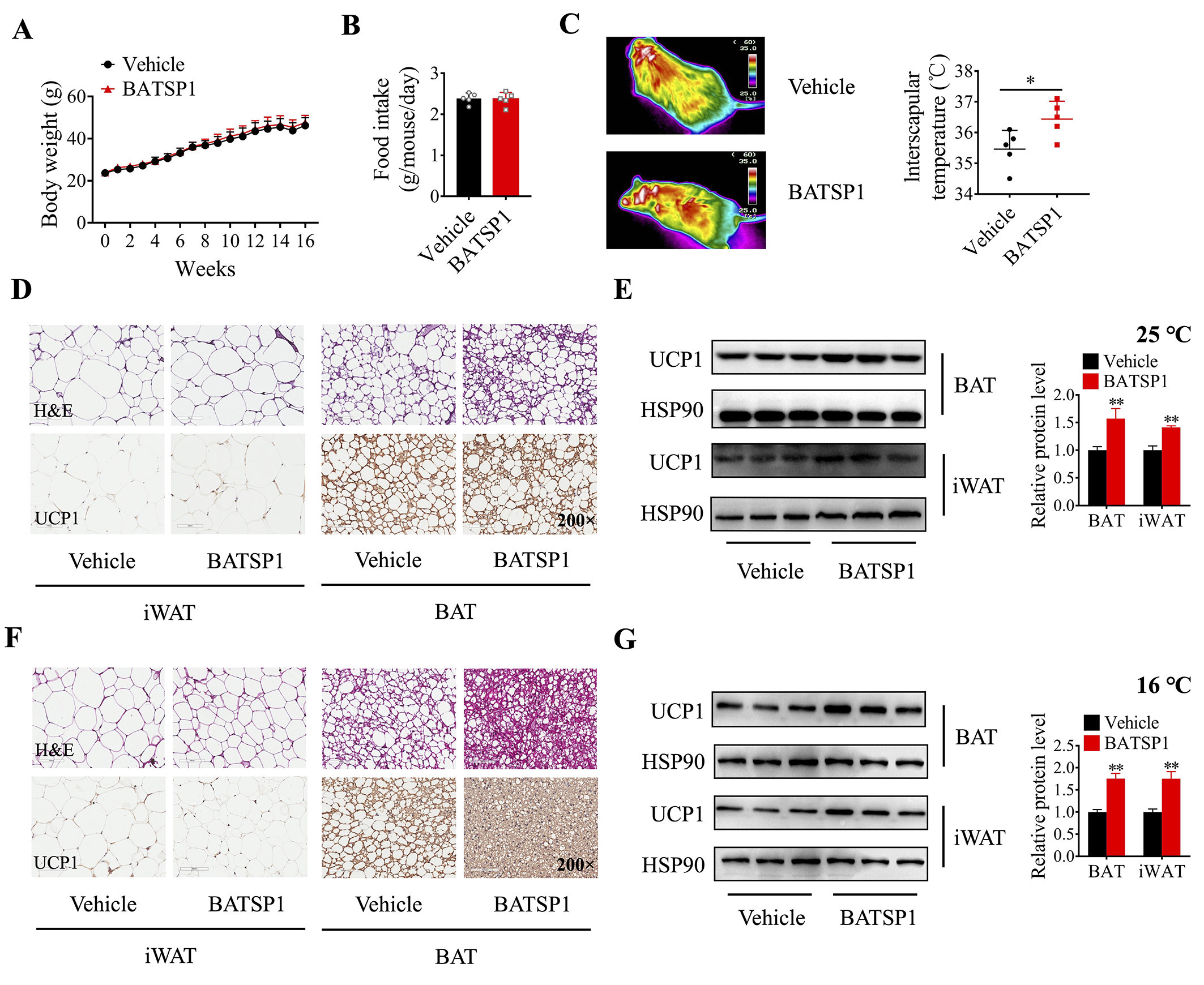
**

**Fig. S5 BATSP1 enhances thermogenesis in BAT and induces browning of WAT in HFD mice**

BATSP1- and vehicle-treated mice were maintained at 25 °C (A-E, n=10/group) or 16 °C (F and G, n=10/group) and fed an HFD for 16 weeks. (A) Body weight of HFD-fed mice (n=10/group). (B) Daily food intake of mice. (C) Representative infrared images of the vehicle- and BATSP1-treated mice. (D) H&E staining and immunohistochemistry staining for UCP1 in BAT and iWAT sections. (E) Immunoblot analysis of UCP1 in BAT and iWAT. (F) BAT and iWAT from BATSP1- and vehicle-treated HFD mice were sectioned and stained either with H&E (top) for visualization of general morphology or with Anti-UCP1 antibody for immunohistochemical analysis. (G) Representative Western blots showing UCP1 expression changes in BAT and iWAT.

**
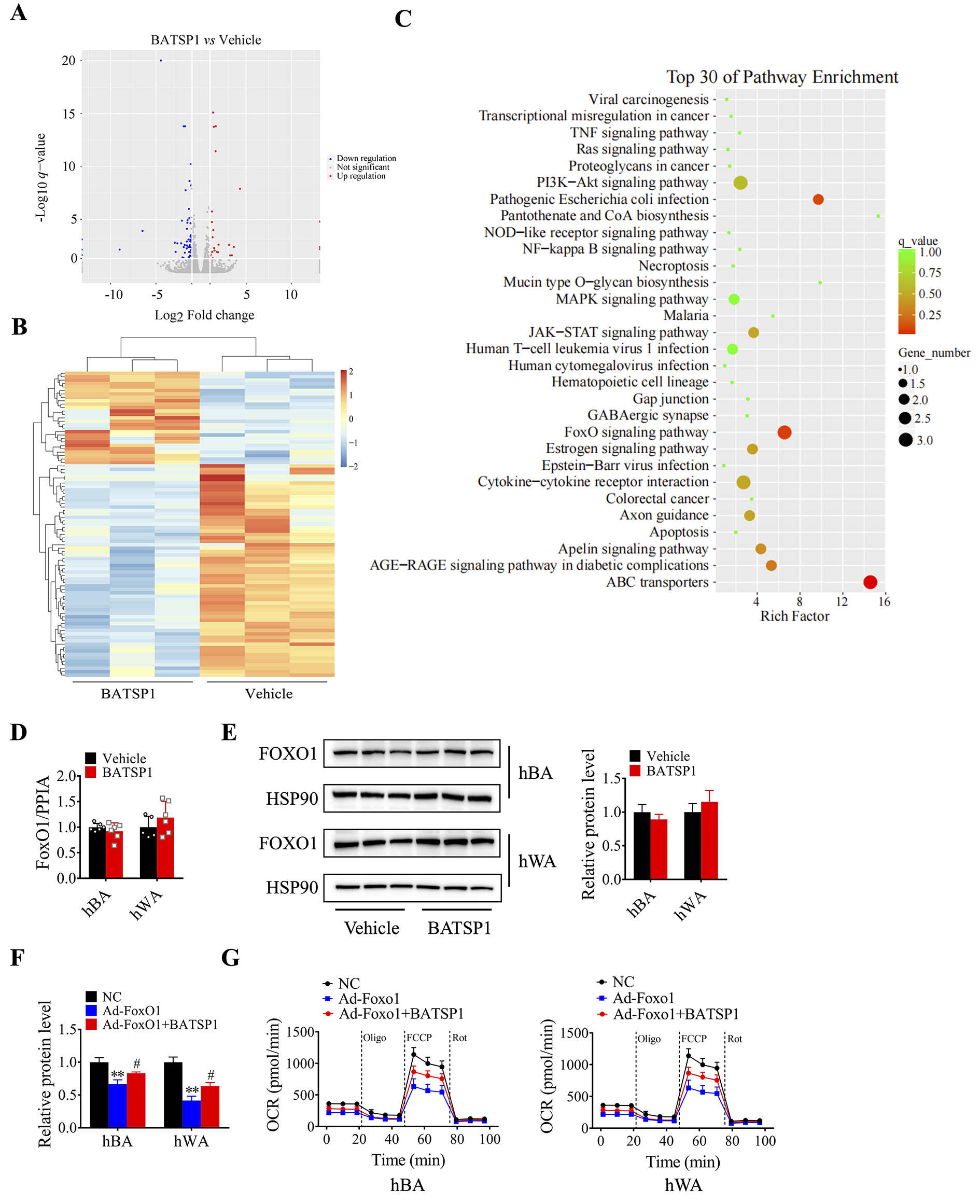
**

**Fig. S6 FOXO1 signaling pathway is involved in BATSP1-mediated thermogenesis of adipocytes**

Human brown adipocytes were treated with BATSP1 for 6 h, and samples were collected for RNA-Seq analysis. (A) The scatter plot in the log2 scale indicates the fold changes of gene expression in BATSP1-treated brown adipocytes compared with vehicle control. (B) Heatmap of all differentially regulated genes between BATSP1-stimulated and unstimulated brown adipocytes. (C) KEGG analysis of RNA-seq data showing top-ranked pathways. (D and E) Molecular level changes of FOXO1 in brown and white adipocytes induced by BATSP1. (F) Quantization for the Western blot results of UCP1. (G) Traces for the Seahorse experiments in adipocytes.

**
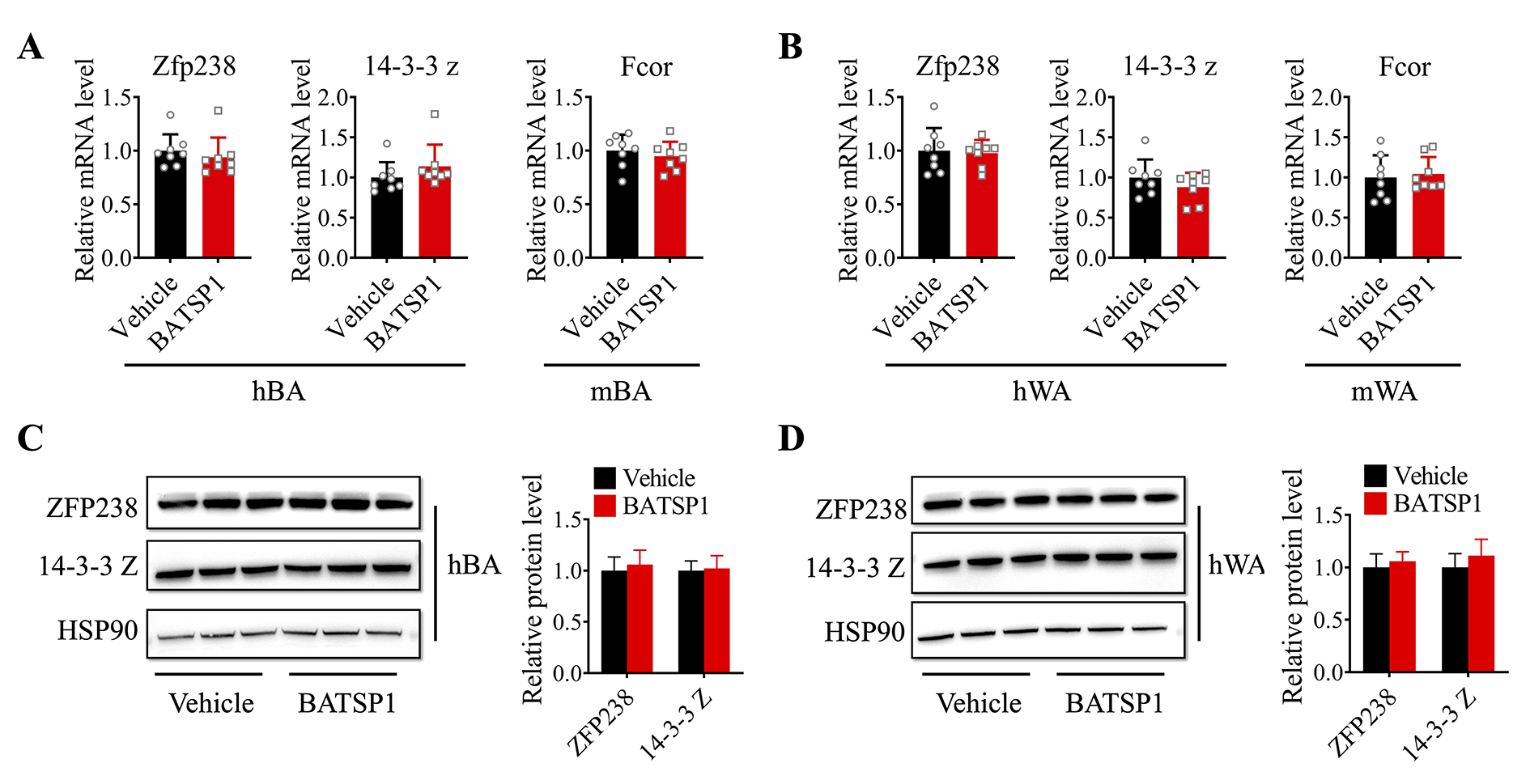
**

**Fig. S7 Expression of genes that are implicated in FOXO1-mediated repression.**

Primary brown and white adipocytes from humans or mice were isolated and induced adipogenic differentiation. Mature adipocytes were treated with BATSP1 for 6 h, and samples were collected for further analyses. (A and B) Transcription levels of Zfp38, 14-3-3 Z and FCoR were determined by RT-qPCR. (C and D) Protein expression levels of Zfp238 and 14-3-3 Z were determined by Western blot.
